# The Geometry of the Bone Structure Associated with Total Hip Arthroplasty

**DOI:** 10.1371/journal.pone.0091058

**Published:** 2014-03-07

**Authors:** Zhang Yang, Wang Jian, Li Zhi-han, Xiao Jun, Zhao Liang, Yan Ge, Shi Zhan-jun

**Affiliations:** Department of Orthopaedic Surgery, Nanfang Hospital, Southern Medical University, Guangzhou, Guangdong, China; Faculty of Animal Sciences and Food Engineering, University of São Paulo, Pirassununga, SP, Brazil, Brazil

## Abstract

Close adaptation of the prosthesis to the bone is the key to achieving optimal stability and fixation for total hip arthroplasty (THA). However, there have been no adequate studies of bone morphology, especially in different races. The aim of this study was to analyze the geometry of the acetabulum and proximal femur of people from South China, based on three-dimensional reconstruction, and to detect differences between different population subsets. CT scans were performed on 80 healthy volunteers (160 hips) from South China, comprising 40 males (80 hips) and 40 females (80 hips). The images were imported into Mimics 10.01 to perform 3D reconstruction. THA-associated anatomical parameters were measured and compared with other published data. In comparison with published data, it seemed that people from South China have smaller acetabular abduction angle, larger acetabular supro-inferior diameter, larger neck-shaft angle, smaller offset, thinner femoral shaft and more proximal isthmus, which needed to be further confirmed. There were significant differences between the genders in most parameters. As significant differences in canal flare index (CFI) and distal canal flare index (DCFI) were found between genders, it was concluded the most significant differences lay in the isthmus of the femur. Among the femora, according to Noble’s classification we identified more normal types and fewer stovepipe and champagne-flute types than expected from the literature, indicating that uncemented prostheses would be suitable for most people from South China. Our findings reveal that simply choosing the smallest of a series of prostheses would not necessarily provide a good fit, due to the different trends from the proximal to the distal part of the femur. Significant variation exists in THA-associated anatomy between genders and population subsets. It is therefore imperative that each patient receives individual consideration rather than assuming all patients have the same anatomy, especially for different races.

## Introduction

Total hip arthroplasty (THA) has been proven to be a good choice for many disorders of the hip [1]. During development of hip prostheses, a variety of implants have been designed for THA. As a result a consensus has been reached among surgeons that close adaptation of the prosthesis to the bone geometry is required to achieve optimal primary stability and secondary biologic fixation [2]. Therefore, the key to successful THA is sound knowledge of the geometry of the associated bone structures, the acetabulum and proximal femur. The geometry of the bone varies from individual to individual, thus implants are made in a range of sizes to match bone geometry. However, these prostheses would fit only common anatomies, rather than taking into account variations between different genders, ages and races [3]. Despite the importance of morphological study, no adequate research has been carried out to investigate the shape of the bone, especially the endosteal geometry. On the contrary, it has been reported that periosteal femoral geometry should be used to design the implant, because of its association with the medullary cavity [4].

As part of the development of surgical technique, a series of related studies have been carried out. Noble *et al.* [4] demonstrated the presence of both endosteal and periosteal variation, and the need for multiple stem designs to achieve close fit. Walker and Robertson [5] designed a femoral stem to fit an average femoral cavity based on a three-dimensional analysis, and observed considerable variation in the shape and angulation of the femoral cavity at the osteotomy level. Husmann *et al.* [6] described the endosteal morphology of the proximal femur focusing on the variation of the metaphyseal region, implying that a single cementless stem design, even if several sizes were available, would not fit all femoral cavities [7].

Yet, despite the published data, there are no adequate data on bone morphology, especially in different races. We found that, in our clinical practice using imported prostheses, the two adjacent sizes of prosthesis may be too large and too small, or in some cases the distal component may be suitable while the proximal component was too small. We hypothesized that the THA-associated geometry was different between genders, and that of people from South China, especially enlargement of the endosteal dimensions, differed significantly from the Caucasian model according to which the implants were designed. The aims of this study were to (1) investigate and describe the geometry of the acetabulum and proximal femur of people from South China, (2) investigate variations in the acetabulum and proximal femur among different individuals, and (3) determine whether the sizes of the acetabulum and proximal femur were related to demographic data, such as gender and age, based on three-dimensional (3D) reconstruction which was a more credible method.

## Materials and Methods

### Selection of Study Population

This study was approved by the ethics committee of Nanfang Hospital and signed informed consent was obtained from each patient. In this study, the included population was a nonrandomized, healthy group of volunteers. The inclusion criteria were as follows: people from Guangdong and Guangxi province of China (whose last five generations also lived in the two provinces), having no hip disorders. Pregnant women and people with the following signs of the hip were excluded: pain, deformity, abnormal movement, claudication, rheumatic fever, rickets, rheumatoid arthritis, osteoarthritis, osteonecrosis of the femoral head, fracture or previous surgery.

Eighty individuals (40 males and 40 females) were recruited in this study. One hundred and sixty scanned hips were available for analysis. The average age was 31.38 (20–45) years, the average height was 167.25 (151–185) cm, and the average weight was 59.99 (40–80) kg. The procedures followed were in accordance with the Helsinki Declaration of 1975, as revised in 2000.

### CT Evaluation

All patients were scanned following an identical protocol using a 64-slice multi slice spiral CT (GE Crop. Fairfield, CT). Patients were placed in the supine position. The lower limbs were fully extended, fixed in neutral rotation with toes pointed up, and strapped to prevent movements during acquisition. CT scans were taken with a contiguous thickness of 0.625 mm, from the anterior superior spine to the top of the patella, with settings of 120 kV and 80 mA. Images from each CT scan were saved as DICOM images and recorded on a separate CD-ROM.

### 3D Reconstruction and Measurement

The DICOM images were imported into Mimics 10.01 software (Materialise, Leuven, Belgium) to perform 3D reconstruction. The bone geometry was calculated automatically based on radiodensity and stored as a contour map. The contours from sequential images were connected using triangular surface tiles to create 3D models, which could then be displayed in any position or orientation. Using the software, the following steps were performed to create 3D bone reconstructions: contrast adjustment (0–350), thresholding (226–1821), region growing, editing masks, editing masks in 3D, calculating polylines, cavity filling, boolean operations, morphology operations and 3D calculations. Images in the horizontal, coronal and sagittal planes were defined. The center of the acetabulum and femoral head rotation was deduced in the software by fitting a sphere through the surface of the femoral head.

The following parameters were measured: anteversion angle (AAVA), abduction angle (AABA) and supro-inferior diameter (ASD) of the acetabula; the femoral anteversion angle (FAVA), neck-shaft angle (NSA), diameter of the femoral head (FHD), offset, medial-lateral diameter and antero-posterior diameter of the bone medullary cavity at a plane 20 mm above (MLD+20,APD+20), at the midpoint (MLD,APD), and 20 mm below the lesser trochanter (MLD−20, APD−20), the internal (IID) and external diameter (IED) of the isthmus, and the position of the isthmus (IP). Canal flare index (CFI) was calculated as the ratio between the medial-lateral canal width 20 mm above the midpoint of the lesser trochanter and the isthmus width [4]. Metaphyseal canal flare index (MCFI) was determined to specify the variability of the proximal femoral opening (the ratio between mediolateral canal width 20 mm above and 20 mm below the midpoint of the lesser trochanter). Distal canal flare index (DCFI) was defined as the ratio between mediolateral canal width 20 mm below the midpoint of the lesser trochanter and isthmus width, to specify the opening of the femoral diaphysis.

To perform the above measurement, several key points need to be located. Spherical fitting was performed to the femoral head, and the center of the sphere was regarded as the center of the femoral head. The axes of the femoral shaft and neck were calculated as the center of polylines of the endosteal cavity, and the intersection of the two axes was regarded as the break point between the shaft and neck. Other points were located according to their coordinates along certain axes, which meant the points with minimum or maximum coordinates were chosen. For example, when measuring the internal diameter of the isthmus, all coordinates of points on the medullary cavity were exported, and the two points with maximum and minimum coordinates along the X axis were chosen.

### Statistical Analysis

The intraclass correlation coefficient (ICC) was applied to assess the reliability: 0.00 to 0.20, poor; 0.21 to 0.40, fair; 0.41 to 0.60, moderate; 0.61 to 0.80, substantial; and 0.81 to 1.00, perfect. In order to ensure the reliability of the results, 40 cases (20 males and 20 females) were selected randomly, and two authors performed the measurements on these cases twice each with an interval of one month.

Distribution of the values was assessed using descriptive statistical analysis. Normally-distributed data were compared using Student’s *t* test, otherwise a non-parametric test was applied. Pearson correlations were used to determine the relationships between different anatomic parameters. In correlation analysis, the data of only one side was selected randomly. Results of different studies were compared by ANOVA. *P*-values <0.05 were considered significant.

## Results

Measurement reliability was satisfactory with ICC varying from 0.72–0.93 for different indicators. The measurement results were characterized by a normal distribution after normality testing. The acetabulum was anteverted 20.09° (2.56°) and abducted 49.32° (3.77°), with an average supro-inferior diameter of 58.74 mm (4.20 mm). These findings are summarized in Table 1. The means and standard deviations (SDs) of the dimensions at different levels of the proximal femur are presented in Table 2. There were significant differences in most parameters between males and females.

**Table 1:** Results of measurement of the acetabula of healthy adults in South China.

	Total	Male	Female
AAVA (°)	20.09±2.56	19.82±2.50	20.35±2.62
AABA (°)	49.32±3.77	50.79±3.15	47.85±3.79*
ASD (mm)	58.74±4.20	61.47±3.50	56.03±2.89*

AAVA: acetabular anteversion angle, AABA: acetabular abduction angle, ASD: acetabular suprainferior diameter.

*There was significant difference between the male and the female (P<0.05).

**Table 2:** Results of measurement of dimensions at different levels of the proximal femur and femoral indices.

	Total	Male	Female
FAVA (°)	16.47±2.72	15.52±2.51	17.41±2.61*
NSA (°)	130.24±4.34	129.98±3.66	130.49±4.93
FHD (mm)	45.22±3.61	48.06±1.97	42.37±2.43*
Offset (mm)	37.16±3.85	39.33±3.18	35.00±3.20*
MLD+20 (mm)	41.69±4.43	43.12±4.72	40.26±3.61*
APD+20 (mm)	31.00±3.29	32.03±3.39	29.98±2.86*
MLD (mm)	25.62±4.02	25.61±4.10	25.63±3.96
APD (mm)	23.91±3.46	24.69±3.41	23.13±3.35*
MLD−20 (mm)	19.78±2.43	20.21±2.45	19.36±2.35*
APD−20 (mm)	17.50±3.05	18.19±2.60	16.80±3.31*
IID (mm)	11.34±1.68	12.10±1.37	10.58±1.62*
IED (mm)	25.50±2.32	26.48±2.10	24.52±2.11*
IP (mm)	106.60±5.61	106.93±6.24	106.26±4.90
CFI	3.74±0.57	3.60±0.49	3.88±0.60*
MCFI	2.12±0.24	2.15±0.26	2.10±0.22
DCFI	1.56±0.28	1.51±0.21	1.61±0.34*

FAVA: femoral anteversion angle, NSA: neck-shaft angle, FHD: diameter of the femoral head, Offset: femoral offset, MLD+20 and APD+20: medial-lateral diameter and antero-posterior diameter of bone medullary cavity at a plane of 20 mm above the lesser trochanter, MLD and APD: medial-lateral diameter and antero-posterior diameter of bone medullary cavity at the midpoint of the lesser trochanter, MLD−20 and APD−20: medial-lateral diameter and antero-posterior diameter of bone medullary cavity at a plane of 20 mm below the lesser trochanter, IID: internal diameter of the isthmus, IED: external diameter of the isthmus, IP: distance from the midpoint of the lesser trochanter to the isthmus. CFI: Canal flare index. MCFI: Metaphyseal canal flare index. DCFI: Distal canal flare index.

*There was significant difference between the male and the female (P<0.05).

Several studies have been performed to analyze the bone structure (Table 3 and Table 4). Through comparison with previously-reported findings we conclude that people from South China who were the subjects of this study have a larger neck-shaft angle, smaller offset, thinner shaft and more proximal isthmus. The abduction angle of the acetabulum was smaller and the supro-inferior diameter was larger, while there was no significant difference in the anteversion angle. Taking into the consideration of different methodologies, the comparison need to be further confirmed.

**Table 3:** Comparison of acetabular measurements between this study and other published data.

Author	Tallroth [23]	Vandenbussche [27]	Murphy [28]
Country	Finland	NR	U.S.A
Measuring method	CT	CT reconstruction	CT reconstruction
Sample size	40	100	34
AAVA (°)	21.00±7.00	16.90±5.50*	20.40±7.10
*P*	0.30	0.00	0.73
AABA (°)		38.70±4.80*	53.00±6.30*
*P*		0.00	0.00
ASD (mm)		48.50±4.40*	
*P*		0.00	

AAVA: acetabular anteversion angle, AABA: acetabular abduction angle, ASD: acetabular suprainferior diameter.

*Significantly different compared with our study (*P*<0.05).

NR: not reported.

**Table 4:** Comparison of femoral measurements between this study and other published data.

Author	Massin [2]	Blaimont [3]	Rubin [25]	Noble [4]	Laine [7]
Country	France	NR	NR	U.S.A	NR
Measurement method	X-ray	X-ray	Cadaver	Cadaver	CT of cadaver
Sample size	400	166	32	200	50
NSA(°)	123.1±8.2*	124±8*	122.9±7.6*	124.7±7.4*	
*P*	0.00	0.00	0.00	0.00	
FHD (mm)	45.6±4.2		43.4±2.6*	46.1±4.8	
	0.45		0.01	0.14	
Offset (mm)	41.0±6.2*	46.6±7*	47.0±7.2*	43.0±6.8*	
		0.00	0.00	0.00	
MLD+20 (mm)	44.1±6.0*		43.1±5.0	45.4±5.3*	45.42±4.46*
			0.15	0.00	
APD+20 (mm)					31.39±3.45
*P*					0.52
MLD (mm)	26.5±3.6		27.9±3.6*	29.4±4.6*	28.73±3.21*
	0.05		0.01	0.00	
APD (mm)					25.58±2.86*
*P*					
			21.0±2.7*	20.9±3.5*	20.41±2.14
			0.02	0.01	0.14
APD−20 (mm)					20.71±2.47*
*P*					0.00
IID (mm)	12.4±2.3*	14.8±3*	13.1±2.1*		11.06±1.88*
*P*	0.00	0.00	0.00		
IED (mm)	27.6±3.0*		26.7±1.8*		
*P*	0.00		0.00		
IP (mm)			105.7±17.9	113.4±16.4*	
*P*			0.69	0.00	
CFI	3.6±0.8		3.36±0.75*		
*P*	0.06		0.01		

NSA: neck-shaft angle, FHD: diameter of the femoral head, MLD+20 and APD+20: medial-lateral diameter and antero-posterior diameter of bone medullary cavity at a plane of 20 mm above the lesser trochanter, MLD and APD: medial-lateral diameter and antero-posterior diameter of bone medullary cavity at the midpoint of the lesser trochanter, MLD−20 and APD−20: medial-lateral diameter and antero-posterior diameter of bone medullary cavity at a plane of 20 mm below the lesser trochanter, IID: internal diameter of the isthmus, IED: external diameter of the isthmus, IP: distance from the midpoint of the lesser trochanter to the isthmus. CFI: Canal flare index.

*Significantly different compared with our study (*P*<0.05).

NR: not reported.

Considering the correlation between diameters at different levels of the proximal femur with age and height (Table 5), the correlation coefficients varied from −0.010 (internal diameter of isthmus *vs.* offset, *P = *0.908) to 0.833 (internal diameter of isthmus *vs.* medial-lateral diameter of bone medullary cavity 20 mm above lesser trochanter, *P = *0.000). Weaker correlations were generally observed between most of the parameters. Significant correlations were detected between variables describing the canal width in the vicinity of the lesser trochanter, consistent with the studies of Dai *et al.* [8] and Noble *et al.* [4]. The dimensions of the femoral neck did not correlate or correlated weakly with the size of the femur (Table 6). There was no strong correlation between the dimensions of the femoral neck and the acetabulum (Table 7).

**Table 5:** Correlation between diameters at different levels of the proximal femur with age and height.

e	MLD+20	APD+20	MLD	APD	MLD−20	APD−20	IID	IED	IP	CFI	MCFI	DCFI
Age	0.230*	0.063	−0174*	0.049	0.142	−0.064	−0.153*	0.193	0.065	−0.075	−0.122	−0.067
Height	0.157	0.091	−0.195*	−0.110	0.068	−0.040	−0.098	0.146	−0.200	−0.102	−0.026	−0.112
MLD+20		0.132	0.253*	−0.099	−0.104	0.046	0.833*	−0.187*	−0.134	0.937*	0.981*	−0.073
APD+20			0.258*	0.367*	0.080	−0.091	−0.141	0.253*	−0.122	−0.081	0.048	−0.084
MLD				0.105	0.391*	−0.113	−0.122	0.022	0.069	−0.148	−0.024	−0.009
APD					0.169*	0.223	0.175*	−0.237*	0.072	0.127	0.075	0.054
MLD−20						0.290*	0.224*	0.072	−0.159	0.147	−0.979*	0.070
APD−20							0.059	−0.045	−0.045	0.038	−0.012	0.978*
IID								0.211*	0.241	−0.973*	0.060	−0.970*
IED									0.084	0.186*	0.050	0.163
IP										0.248*	−0.046	0.181
CFI											−0.020	0.770*
MCFI												−0.242*

MLD+20 and APD+20: medial-lateral diameter and antero-posterior diameter of bone medullary cavity at a plane of 20 mm above the lesser trochanter, MLD and APD: medial-lateral diameter and antero-posterior diameter of bone medullary cavity at the midpoint of the lesser trochanter, MLD−20 and APD−20: medial-lateral diameter and antero-posterior diameter of bone medullary cavity at a plane of 20 mm below the lesser trochanter, IID: internal diameter of the isthmus, IED: external diameter of the isthmus, IP: distance from the midpoint of the lesser trochanter to the isthmus. CFI: Canal flare index. MCFI: Metaphyseal canal flare index.

*P<0.05.

**Table 6:** Correlation between dimensions of the femoral neck and femoral sizes.

	FAVA	NSA	FHD	Offset
Age	0.179*	0.252*	0.144	0.150
Height	0.247*	0.183*	0.532*	0.247*
FAVA		−0.039	−0.167*	−0.100
NSA			−0.107	−0.094
FHD				−0.094
offset				
MLD+20	−0.201*	0.120	−0.072	0.105
APD+20	0.067	−0.269*	0.003	0.171*
MLD	0.244*	0.056	0.089	−0.175*
APD	−0.043	0.162	0.095	−0.125
MLD−20	−0.032	−0.026	0.058	0.031
APD−20	0.145	−0.113	0.164*	0.050
IID	0.157	−0.080	0.156	−0.010
IED	−0.058	0.038	0.006	0.066
IP	0.060	−0.120	0.007	0.037

FAVA: femoral anteversion angle, NSA: neck-shaft angle, FHD: diameter of the femoral head, MLD+20 and APD+20: medial-lateral diameter and antero-posterior diameter of bone medullary cavity at a plane of 20 mm above the lesser trochanter, MLD and APD: medial-lateral diameter and antero-posterior diameter of bone medullary cavity at the midpoint of the lesser trochanter, MLD−20 and APD−20: medial-lateral diameter and antero-posterior diameter of bone medullary cavity at a plane of 20 mm below the lesser trochanter, IID: internal diameter of the isthmus, IED: external diameter of the isthmus, IP: distance from the midpoint of the lesser trochanter to the isthmus.

*P<0.05.

**Table 7:** Correlation between dimensions of the femoral neck and the acetabulum.

	AAVA	AABA	ASD	FAVA	NSA	FHD	Offset
AAVA		0.142	−0.100	−0.156	0.026	−0.104	0.030
AABA			−0.209*	0.071	0.021	0.019	−0.002
ASD				−0.031	0.097	0.206*	−0.114

AAVA: acetabular anteversion angle, AABA: acetabular abduction angle, ASD: acetabular suprainferior diameter.

FAVA: femoral anteversion angle, NSA: neck-shaft angle, FHD: diameter of the femoral head.

*P<0.05.

## Discussion

In addition to relieving pain, the objective of THA is to achieve good reconstruction of the hip [9] and thus diminish the risk of future degenerative changes and dislocation, accomplished by reorienting the acetabulum to achieve improved loading conditions at the joint surface [10]. A series of clinical and experimental studies of THA have demonstrated that it is essential to achieve a close geometric fit between the prosthetic component and the supporting bone for primary and durable fixation. Malposition of the acetabular cup results in increased risk of dislocation, limited range of motion, and impingement [11]. The stability of the femoral component depends on a balance of proximal and distal load transfer from the implant to the femur [12]. The relative contributions of the proximal and distal support depend mainly on the fit of the prosthesis to the bone. Experiments have suggested that relative motion between the implant and bone more than 14 micra before bone ingrowth will lead to inadequate implant fit and fibrous encapsulation of the implants. Thus to achieve a satisfactory outcome of THA, detailed knowledge of the associated anatomy is required [4].

### Anatomy in Different Races

**Figure 1:**
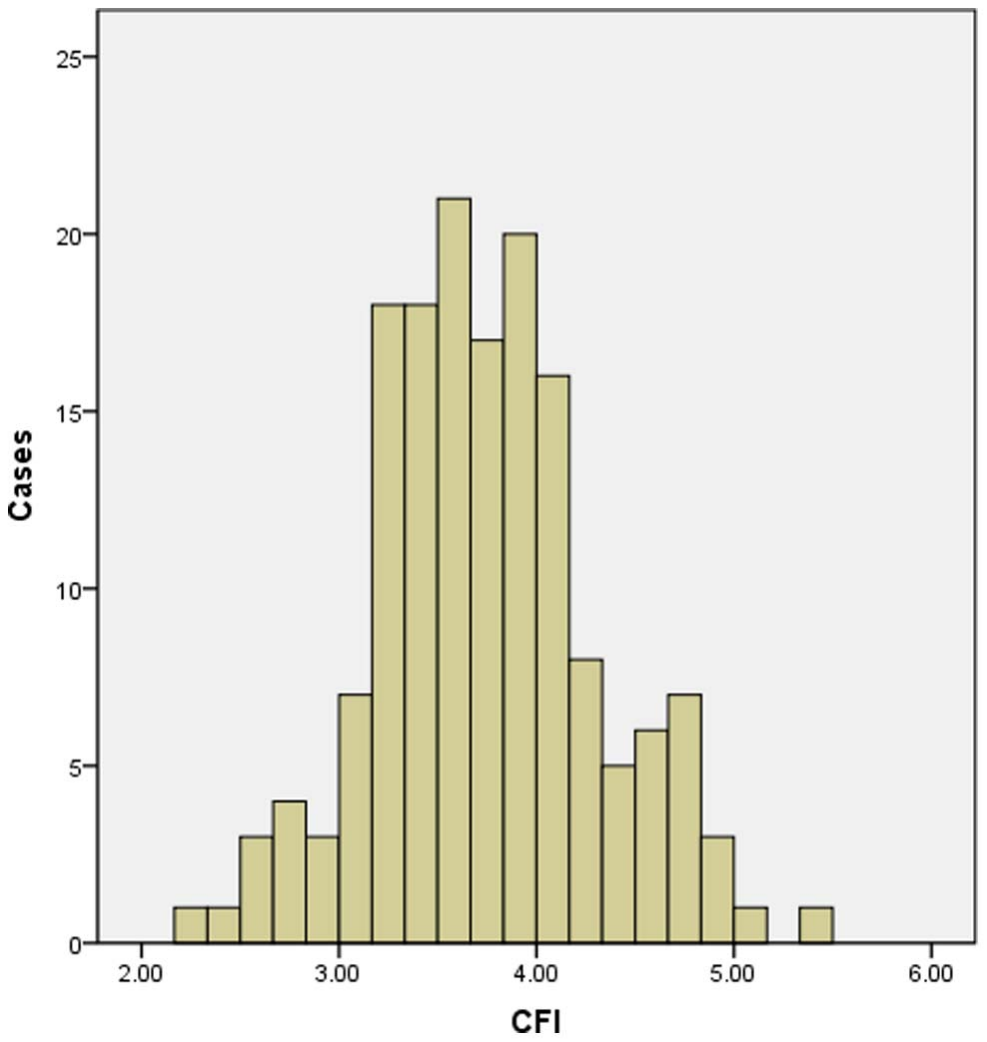
Distribution of the CFI of the proximal femur.

Given the wide individual variability in THA-associated anatomical bone structure, it should be noted that one procedure/implant could cure one patient’s hip disorders but worsen another’s. Small changes during osteotomy or in implant design could either drastically improve or worsen the outcome of the procedure. The variability in bone morphology is consistent with the consensus that the geometry of the femur is determined by a series of genetic and environmental factors, such as race, sex, age, and life-style. These influences appear to lead to unique bone geometries, as characteristic of each individual as any other feature of human anatomy [4]. Based on the classification of CFI described by Noble *et al.* [4], the shape distribution of the femoral cavity is shown in Figure 1. Compared with the report by Noble *et al.*, there were more normal types (85% *vs*. 83%) but fewer stovepipe types (7.5% vs. 9%) or champagne-flute types (7.5% *vs*. 8%).

Good clinical results and midterm survival rates of cementless THA have been published [13], but failures of prosthetic design still occur, even in the short-term [14], caused by micromotion of the prosthesis. An implant which perfectly fills both the proximal and distal femoral canal would be difficult to insert without breaking the femur [15]. Finite-element studies [16] and photoelastic coating strain analysis [5] have demonstrated the importance of metaphyseal fit in achieving physiologic-like implant–bone load transfer and in minimizing stress shielding and disadvantageous bone remodeling. Femoral stem micromotion studies have also emphasized the importance of metaphyseal endosteal stem fit in the reduction of torsional motion [17]. Thus a good metaphyseal fit is considered to be one of the major goals in cementless femoral stem design. With this objective in mind, the femoral stem should be selected according to the metaphyseal shape, which is described by MCFI rather than CFI, which depicts the entire proximal bone. This is also demonstrated in this study, in which MCFI was found to correlate with the medullary cavity width 20 mm above and below the lesser trochanter, while CFI correlates with the medullary cavity width 20 mm above the lesser trochanter and the dimensions of the isthmus. Thus, it was found that simply choosing the smallest size of a series of prostheses would not provide a good fit, due to the different trends from the proximal to the distal part of the femur.

Surgeons are always trying to restore the original position of the center of the femoral head to ensure maintenance of leg length and reestablishment of the original balance joint forces. Thus femoral components must be available in a range of neck lengths (neck-shaft angle, offset) for each separate stem size. Improving the design of the epiphyseal section of the implant becomes a necessity. Observations in this study showed variation of the neck shaft angle of 27.53°, which is consistent with the results of Clark *et al.* [18]. Neck shaft angle did not correlate with the size of the femur, implying that it would be logical to propose several neck shaft angles for each stem [2]. Custom-made [19] and modular [20] femoral prostheses have been designed to overcome such problems. Early radiologic and clinical results using modular prostheses have been promising, but some problems, for example intraoperative femoral fractures [21], still remain to be overcome. However poor results have also been reported with nonporous custom-made components [22].

### Gender Differences

It was noteworthy that, in this study as well as those discussed above, gender differences existed in most of the parameters, and were of the same magnitude, implying that surgeons should take the slight variations between male and female geometry into account when planning and performing THA. The results highlight the importance of determining normal values for the acetabular orientation of the different genders [23]. Furthermore, as the CFI and DCFI differ significantly between the two genders, we found that the most significant differences lay in the isthmus of the femur. Some knee implants have been designed to be gender-specific ostensibly to provide a better anatomic fit and improved bone coverage. Such implants are expected to be developed for use in THA to reduce the incidence of post-operative dislocation.

### Measurement Methods

Anatomic measurements based on CT images are reported to be convenient and rapid for routine diagnosis, as well as for preoperative analysis if necessary [24]. Additionally, CT evaluations provide surgeons with the information they need to choose suitable candidates for surgery. The ability to define the goals of surgery and to plan appropriate adjustments are of paramount importance. Also, it can be concluded from our study that CT reconstruction is a precise but expensive investigation which is not always easily available to the surgeon. Conventional radiographs provide an acceptable level of accuracy in the distal femur, but not the proximal. Their imprecision is increased in clinical practice by other factors, such as incorrect positioning of the patient because of pain or contracture, or small variations in leg rotation, which significantly alter neck-shaft angle and isthmus width [25]. Conventional 2D radiology thus remains the most convenient method available for routine THA. However, the absence of precise radiological data may result in insufficient accuracy to perform pre-operative design for custom-made prostheses.

The application of 3D reconstruction in studies has numerous advantages over cadaver specimens: greater number of cases, accessible demographic data, ideal magnification and spatial orientation of the bone structure. By means of digitization, points can be clearly marked and their placement may be corrected a posteriori, if needed.

The characteristics of this study can be summarized as aiming at different races and precision of measurements. The availability of THA-associated geometric data allows guidelines to be developed for the procedure and assessment of the match between the bone and the prosthesis. It is imperative that each patient should be considered individually even while assumed to have the same anatomy [26], especially for different races. Failure to understand individual characteristics may lead to incorrect surgical performance.
